# Deafness-associated mitochondrial 12S rRNA mutation reshapes mitochondrial and cellular homeostasis

**DOI:** 10.1016/j.jbc.2024.108124

**Published:** 2024-12-22

**Authors:** Yunfan He, Zhining Tang, Gao Zhu, Luhang Cai, Chao Chen, Min-Xin Guan

**Affiliations:** 1 Center for Mitochondrial Biomedicine and Department of Otolaryngology-Head and Neck Surgery, The Fourth Affiliated Hospital, Zhejiang University School of Medicine, Yiwu, Zhejiang, China; 2 Institute of Genetics, Zhejiang University International School of Medicine, Hangzhou, Zhejiang, China; 3 Center for Genetic Medicine, Zhejiang University International Institute of Medicine, Yiwu, Zhejiang, China; 4 Clinical Laboratory, Huzhou Central Hospital, Affiliated Central Hospital of Huzhou University, Huzhou, Zhejiang, China; 5 Joint Institute of Genetics and Genomic Medicine Between Zhejiang University and University of Toronto, Hangzhou, Zhejiang, China

**Keywords:** deafness, 12S rRNA, mitochondrial DNA mutation, mitophagy, intracellular signaling, cellular Integrity, apoptotic pathways

## Abstract

Human mitochondrial 12S ribosomal RNA (rRNA) 1555A>G mutation has been associated with aminoglycoside-induced and nonsyndromic deafness in many families worldwide. Our previous investigation revealed that the m.1555A>G mutation impaired mitochondrial translation and oxidative phosphorylation (OXPHOS). However, the mechanisms by which mitochondrial dysfunctions induced by m.1555A>G mutation regulate intracellular signaling for mitochondrial and cellular integrity remain poorly understood. Here, we demonstrated that the m.1555A>G mutation downregulated the expression of nucleus-encoded subunits of complexes I and IV but upregulated the expression of assemble factors for OXPHOS complexes, using cybrids derived from one hearing-impaired Chinese subject bearing the m.1555A>G mutation and from one hearing normal control lacking the mutation. These alterations resulted in the aberrant assembly, instability, and reduced activities of respiratory chain enzyme complexes I, IV, and V, rate of oxygen consumption, and diminished ATP production. Furthermore, the mutant cell lines carrying the m.1555A>G mutation exhibited decreased membrane potential and increased the production of reactive oxygen species. The aberrant assembly and biogenesis of OXPHOS impacted mitochondrial quality controls, including the imbalance of mitochondrial dynamics *via* increasing fission with abnormal mitochondrial morphology and impaired mitophagy. Strikingly, the cells bearing the m.1555A>G mutation revealed the upregulation of both ubiquitin-dependent and independent mitophagy pathways, evidenced by increasing levels of Parkin, Pink, BNIP3 and NIX, respectively. The m.1555A>G mutation-induced deficiencies ameliorate the cell homeostasis *via* elevating the autophagy process and upregulating apoptotic pathways. Our findings provide new insights into pathophysiology of mitochondrial deafness arising from reshaping mitochondrial and cellular homeostasis due to 12S rRNA 1555A>G mutation.

Mutations in mitochondrial DNA (mtDNA) have been linked to a wide spectrum of clinical presentations, including neuromuscular disorders, diabetes, vision failure, and deafness (1, 2, 3, 4, 5, 6, 7, 8, 9). The mtDNA is a 16,569-bp circular double-strand molecule, encoding 13 polypeptides for oxidative phosphorylation (OXPHOS) complexes, 22 tRNAs, 12S rRNA, and 16S rRNA essential for the mitochondrial translation (1, 10). However, approximately 1500 mitochondrial proteins including subunits of OXPHOS, mitochondrial dynamics and mitophagy are encoded by nuclear genes, translated into the cytosol and transported into mitochondria (2, 11). In particular, mitochondrial RNA mutations have been associated with sensorineural deafness that often occurs as a consequence of damaged or deficient inner ear hair cells (6, 7, 8, 9). The syndromic deafness (deafness with other medical problems such as diabetes)-associated tRNA mutations include the MELAS-associated tRNA^Leu(UUR)^ 3243A>G and MERRF-associated tRNA^Lys^ 8344A>G and maternally inherited diabetes and deafness (MIDD)-associated tRNA^Glu^ 14692A>G mutations (12, 13, 14). The nonsyndromic deafness (deafness is the only obvious medical problem) associated tRNA mutations are the tRNA^Ser(UCN)^ 7445A>G, and 7511T>C, tRNA^His^ 12201T>C, tRNA^Asp^ 7551A>G, tRNA^Ile^ 4295A>G, tRNA^Cys^ 5783C>T, and m.7516delA mutations (15, 16, 17, 18, 19, 20, 21, 22, 23). The 12S rRNA 1555A>G and 1494C>T mutations have been associated with both aminoglycoside-induced and nonsyndromic deafness in many families worldwide (24, 25, 26, 27). The m.1555A>G and m.1494T>C mutations are located at the highly conserved A-site of 12S rRNA in the mitochondrial ribosomes (7, 24, 25, 28, 29). The homologous region of 16S rRNA in *Escherichia coli* is an essential part of the decoding site of the ribosome where the codon–anticodon recognition occurs and is crucial for action for aminoglycosides (28, 29). The m.1555A>G and m.1494C>T mutations create the 1494C-G1555 or 1494U-A1555 base pair at the A-site of 12S rRNA and make the secondary structure of this rRNA more closely resemble the corresponding region of *E. coli* 16S rRNA, thereby altering the bindings for aminoglycosides and protein synthesis (7, 29). Biochemical characterization showed that the m.1555A>G and m.1494C>T mutations led to the impairing mitochondrial translation, diminishing ATP productions, and excessive generation of reactive oxygen species (ROS) responsible for the cochlear dysfunctions (30, 31, 32, 33, 34). In fact, mitochondria have defense pathways to respond to these genetic and environmental stressors and maintain their quality control *via* fusion and fission processes as well as mitophagy (35, 36, 37, 38, 39). In particular, these mitochondria dysfunctions due to mitochondrial RNA mutations may dysregulate the expression of nuclear genes involved in cellular homeostasis such as autophagy and apoptosis through mitochondrial retrograde signal pathways (35, 40, 41, 42, 43). However, the mechanisms underlying 12S rRNA mutations that reshape the mitochondrial and cellular integrity are far less understood.

In this study, we investigated the mechanism by which 12S rRNA 1555A>G mutation regulates the expression of nuclear genes essential for mitochondrial and cellular integrity. Cybrid cell lines were constructed by transferring mitochondria from lymphoblastoid cell lines derived from an affected matrilineal relative in a Han Chinese family bearing the homoplasmic m.1555A>G mutation and from a control individual lacking the mutation into human mtDNA-less (*ρ*°) cell line (30, 31, 44). We investigated whether m.1555A>G mutation regulates the expression of nuclear-encoded subunits of OXPHOS and impacts the assembly and biogenesis of OXPHOS complexes. Furthermore, we assessed whether the instability and dysfunction of OXPHOS system resulting from the m.1555A>G mutation affect mitochondrial quality control processes involving fission and fusion and mitophagy. We then examined how deficiencies induced by the m.1555A>G mutation impact cellular homeostasis through mechanisms such as autophagy and intrinsic apoptosis.

## Result

### Description and derived cell lines of one hearing-impaired Chinese pedigree

One four-generation Han Chinese family harboring the m.1555A>G mutation (WZD92) was described previously (31). In this pedigree, four matrilineal relatives exhibited a profound hearing loss due to administration with aminoglycoside, whereas other members including 22 matrilineal relatives had normal hearing (31). Further analysis showed that the m.1555A>G mutation was present in homoplasmy in all matrilineal relatives but not in other members of this family. Immortalized lymphoblastoid cell lines were derived from one affected subject carrying the m.1555A>G mutation (WZD92 III-14, male, 32 years) and from one genetically unrelated control subject (A23, male, 33 years) lacking the m.1555A>G mutation belonging to the same mtDNA haplogroup B4 (31). The lymphoblastoid cells were enucleated and subsequently fused to a large excess of mtDNA-less human *ρ*^o^206 cells, derived from the 143B.TK^-^ cell line (30, 44). These cybrid clones were isolated by growing the fusion mixtures in the selective DMEM medium, containing BrdU and lacking uridine, and subsequently analyzed for the presence and levels of the m.1555A>G mutation. The results confirmed the absence of the mtDNA mutation in the control clones and its presence of m.1555A>G mutation in homoplasmy in all cybrids derived from the mutant cell line (data not shown). Two cybrids derived from each donor cell line with similar mtDNA copy number and same karyotype were used for the biochemical characterization.

### Dysregulation of nucleus-encoding subunits of OXPHOS

**Figure 1:**
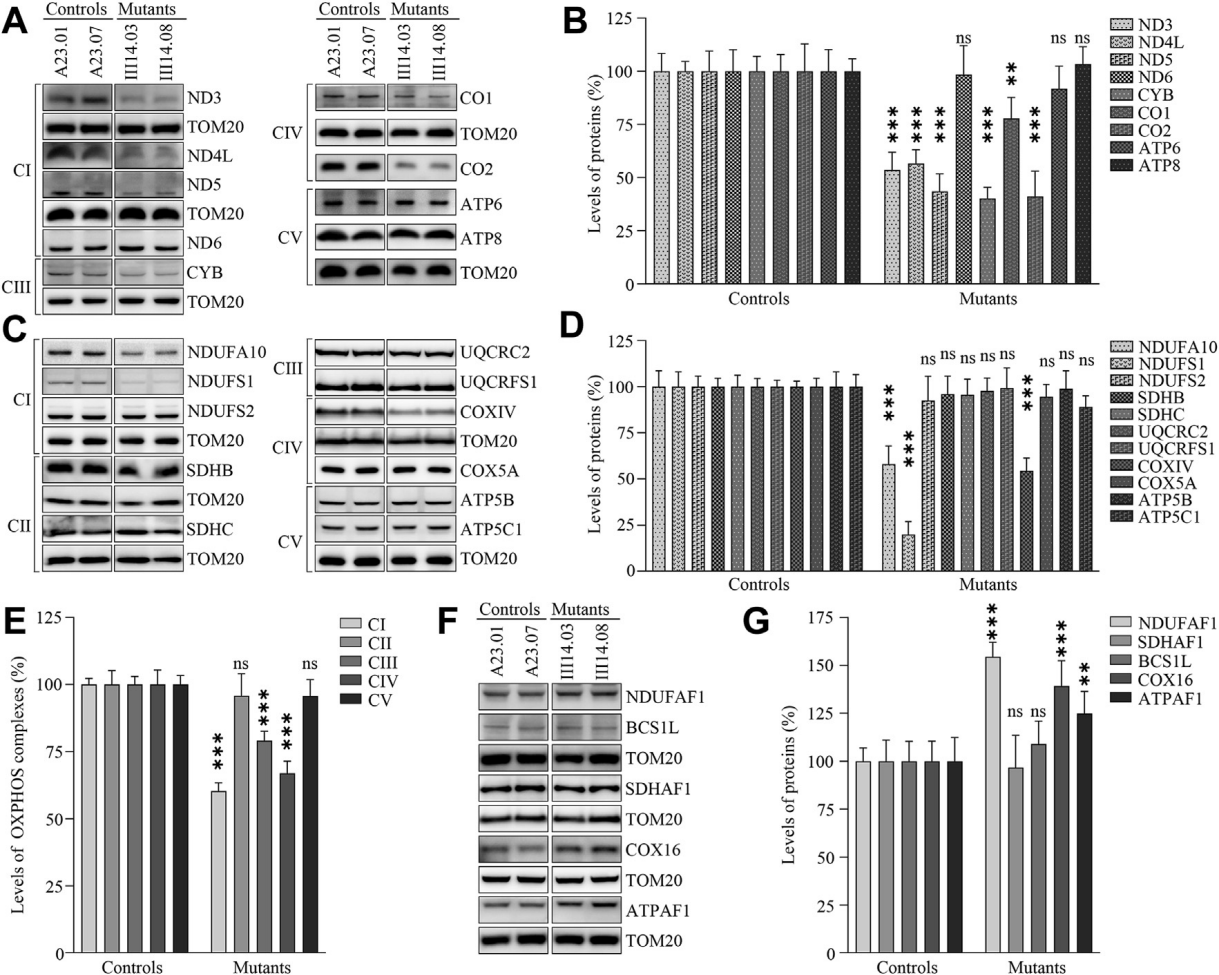
**Western blot analysis of mitochondrial proteins.** Twenty micrograms of total cellular proteins from various cell lines were electrophoresed through a denaturing polyacrylamide gel, electroblotted and hybridized with antibodies for (*A*) Nine mtDNA-encoding subunits [complex I (ND3, ND4L, ND5, ND6), complex III (CYTB), complex IV (CO1, CO3), and complex V (ATP6, ATP8)], (*C*) Eleven nucleus-encoding subunits [complex I (NDUFS1, NDUFS2, NDUFA10), complex II (SDHB, SDHC), Complex III (UQCRC2, UQCRFS1), complex IV (COXIV, COX5A) and Complex V (ATP5B, ATP5C1)], (*F*) Five assembly factors (NDUFAF1, SDHAF1, BCS1L, COX16 and ATPAF1) of OXPHOS complexes, TOM20 as a loading control, respectively. *B, D, E* and *G*, quantification of proteins: (*B*) mtDNA-encoding subunits; (*D*) Nucleus-encoding subunits; (*E*) Average levels of mtDNA and nucleus-encoding subunits of each OXPHOS complexes; (*G*) Assembly factors. Average relative each polypeptide content per cell was normalized to the average content per cell of TOM20 in each cell line. The values for the mutant cell lines are expressed as percentages of the values for the control cell lines. The calculations were based on three independent determinations. The error bars indicate two standard deviations (SD) of the means. *P* indicates the significance, according to the *t* test, of the differences between mutant and control cell lines. ∗*p* < 0.05; ∗∗*p* < 0.001; ∗∗∗*p* < 0.0001; ns, not significant.

To further investigate whether the m.1555A>G mutation impaired mitochondrial translation, we carried out the Western blot analysis to examine the levels in nine mtDNA-encoding polypeptides (ND3, ND4L, ND5, ND6, CYTB, CO1, CO2, ATP6, and ATP8) using two mutant cybrids bearing the m.1555A>G mutation and two cybrids lacking the mutation. As shown in Figure 1*A*, the mutant cybrids harboring the m.1555A>G mutation revealed various levels of these polypeptides, including reductions in the ND3, ND4L, ND5, CYTB, CO1, and CO2, no changes in ND6, ATP6, and ATP8, as compared with these in the control cybrids. As shown in Figure 1*B*, the levels of ND3, ND4L, ND5, ND6, CYTB, CO1, CO2, ATP6, and ATP8 were 53.5%, 56.6%, 43.5%, 98.4%, 40.1%, 77.8%, 41.1%, 91.8%, and 103.3%, respectively, with an average of 67.3% in mutant cybrids, relative to the mean values measured in the control cybrids. These results validated the role of mitochondrial 12S rRNA in mitochondrial translation.

To examine the impact of m.1555A>G mutation on OXPHOS biogenesis, we measured the levels of 11 nucleus-encoding subunits of OXPHOS complexes: NDUFA10, NDUFS1, and NDUFS2 [NADH:ubiquinone oxidoreductase (complex I)], SDHB and SDHC [succinate ubiquinone oxidoreductase (complex II)], UQCRC2 and UQCRFS1 [ubiquinol-cytochrome c reductase (complex III)], COXIV and COX5A [cytochrome C oxidase (complex IV)], ATP5B and ATP5C1 [H+-ATPase (complex V)] (45, 46). As shown in Figure 1*C*, the various reductions in the levels of nine nucleus-encoding subunits were observed in the mutant cybrids, as compared with the control cybrids. As shown in Figure 1*D*, the levels of NDUFA10, NDUFS1, NDUFS2, SDHB, SDHC, UQCRC2, UQCRFS1, COXIV, COX5A, ATP5B, and ATP5C1 were 58.0%, 19.9%, 92.5%, 96.0%, 95.6%, 97.7%, 99.3%, 54.4%, 94.5%, 98.9%, and 88.9%, with an average of 81.4% in the mutant cybrids, relative to the mean values measured in the control cybrids. Notably, the average levels in the subunits of complexes I, II, III, IV, and V in the mutant cybrids were 60.3%, 95.8%, 79.1%, 66.9%, and 95.7% of the average values measured in the control cybrids, respectively (Fig. 1*E*).

We then examined the levels of five assembly factors (NDUFAF1 for complex I, SDHAF1 for complex II, BCS1L for complex III, COX16 for complex IV, and ATPAF1 for complex V) for OXPHOS complexes by Western blot analysis. As shown in Figure 1*F*, the various levels of 5 OXPHOS complex assembly factors were observed in mutant cybrids as compared with those in control cybrids. As shown in Figure 1*G*, the levels of NDUFAF1, BCS1L, COX16, and ATPAF1 were 154.5%, 96.8%, 109.0%, 139.2%, and 124.8%, with an average of 124.9% in mutant cybrids, relative to the mean values measured in the control cybrids.

### Instability and reduced activity of OXPHOS complexes

**Figure 2:**
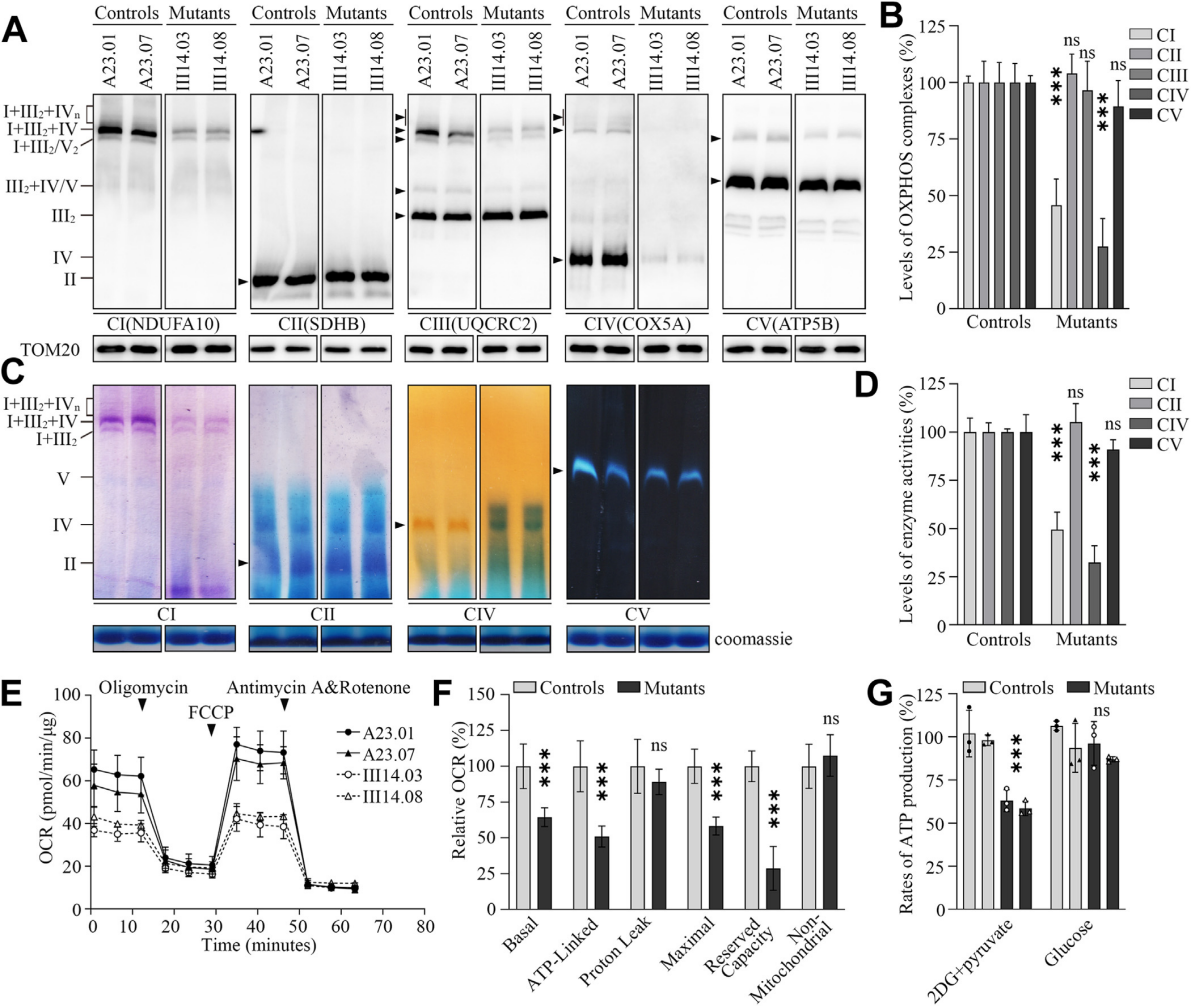
**Analysis of OXPHOS complexes.***A*, the steady-state levels of five OXPHOS complexes. Twenty micrograms of mitochondrial proteins from various cell lines were electrophoresed through BN-PAGE, electroblotted and hybridized with antibodies specific for subunits of five OXPHOS complexes (NDUFS1 for complex I, SDHB for complex II, UQCRC2 for complex III, COX5A for complex IV and ATP5C1 for complex V), and with TOM20 as a loading control. *B*, quantification of levels of complexes I, II, III, IV and V in mutant and control cell lines. *C*, in-gel activities of complexes I, II, IV and V. The activities of OXPHOS complexes from various cell lines after BN-PAGE were measured in the presence of specific substrates for complexes I, II, IV and V, respectively (48). *D*, quantification of in-gel activities of complexes I, II, IV, and V. *E*, analysis of O_2_ consumption with Seahorse Bioscience XF-96 Extracellular Flux Analyzer. The OCR were first measured on 2 × 10^4^ of each cell line under basal condition and then sequentially added to oligomycin (1.5 mM), FCCP (0.5 mM), rotenone (1 mM) and antimycin A (1 mM) at indicated times to determine different parameters of mitochondrial functions. *F*, graphs presented the ATP-linked OCR, proton leak OCR, maximal OCR, reserve capacity, and nonmitochondrial OCR in mutant and control cell lines. *G*, measurement of cellular and mitochondrial ATP levels using bioluminescence assay. ATP levels from mutant and control cell lines were measure using a luciferin/luciferase assay. Mutant and control cell lines were incubated with 10 mM glucose or 5 mM 2-deoxy-D-glucose plus 5 mM pyruvate to determine ATP generation under mitochondrial ATP synthesis. Graph details and symbols are explained in the legend to Figure 1.

We examined the consequence of m.1555A>G mutation on the stability and activities of OXPHOS complexes. Mitochondrial membrane proteins isolated from mutant and control cybrids were separated by blue native polyacrylamide gel electrophoresis (BN-PAGE), electroblotting, and hybridizing with NDUFA10, SDHB, UQCRC2, COX5A and ATP5B, respectively (47, 48). As shown in Figure 2, *A* and *B*, the levels of complexes I, II, III, IV, and V in the mutant cybrids were 45.8%, 103.9%, 96.5%, 27.5%, and 89.4% of those average values in the control cybrids, respectively. These data indicated that the m.1555A>G mutation ablated the assembly of complexes I and IV but not other complexes. The lower levels of the respiratory complexes I, III, IV, and V may be due to the misfolded and/or misassembled of these complexes (46).

We then assessed the consequence of the m.1555A>G mutation on the activity of complexes I, II, IV, and V using the in-gel activity assay (48). Mitochondrial membrane proteins isolated from various cell lines were separated by BN-PAGE and stained in the presence of specific substrates of OXPHOS complexes [NADH and NTB for complex I, sodium succinate, phenazine methosulfate, and NTB for complex II, DAB and cytochrome c for complex IV, glycine, MgSO_4_, ATP, and Pb(NO_3_)_2_ for complex V] (48). Defective assembly of complexes I and IV was further confirmed in the mutant cybrids, as compared with control cybrids (Fig. 2*C*). In particular, the in-gel activities of complexes I and IV in mutant cybrids were 49.4% and 32.4%, relative to the average values of control cybrids, respectively, while the in-gel activities of complexes II and V in the mutant cybrids were comparable with those of control cybrids (Fig. 2*D*). These highlight the impact of the m.1555A>G mutation on the stability and activity of complexes I and IV.

We further investigated the effect of m.1555A>G mutation on respiration using the Seahorse Bioscience XF-96 Extracellular Flux Analyzer. We measured the oxygen consumption rates (OCRs) of various cell lines, including basal respiration, O_2_ consumption attributing to ATP production, proton leak, maximum respiratory rate, reserve capacity, and nonmitochondrial respiration (17, 49). As shown in Figure 2, *E* and *F*, the basal OCR in two mutant cybrids was 64.4% (*p* < 0.001) relative to the mean values measured in two control cybrids. To investigate which of the enzyme complexes of the respiratory chain was affected in the mutant cell lines, oligomycin (to inhibit the ATP synthase), carbonyl cyanide 4-trifluoromethoxy-phenylhydrazone (FCCP) (to uncouple the mitochondrial inner membrane and allow for maximum electron flux through the ETC), rotenone (to inhibit complex I), and antimycin A (to inhibit complex III) were added sequentially while measuring OCR. The difference between the basal OCR and the drug-insensitive OCR yields the amount of ATP-linked OCR, proton leak OCR, maximal OCR, reserve capacity, and nonmitochondrial OCR. As shown in Figure 2, *E* and *F* the ATP-linked OCR, proton leak OCR, maximal OCR, reserve capacity, and nonmitochondrial OCR in two mutant cybrids were 50.9%, 89.1%, 58.3% (*p* < 0.001), 28.6%, and 107.5%, relative to the mean values measured in two control cybrids, respectively.

We then used the luciferin/luciferase assay to examine the capacity of oxidative phosphorylation in mutant and control cybrids. Populations of cells were incubated in the media in the presence of glucose and 2-deoxy-D-glucose with pyruvate (17). As shown in Figure 2*G*, the levels of ATP production in mutant cybrids in the presence of glucose (total cellular levels of ATP) were comparable with those measured in control cybrids. In contrast, the levels of ATP production in mutant cybrids, in the presence of 2-deoxy-D-glucose and pyruvate to inhibit the glycolysis (mitochondrial levels of ATP), varied from 55.7% to 70.0%, with an average of 60.8% relative to the mean values measured in the control cybrids.

### Decreased mitochondrial membrane potential

**Figure 3:**
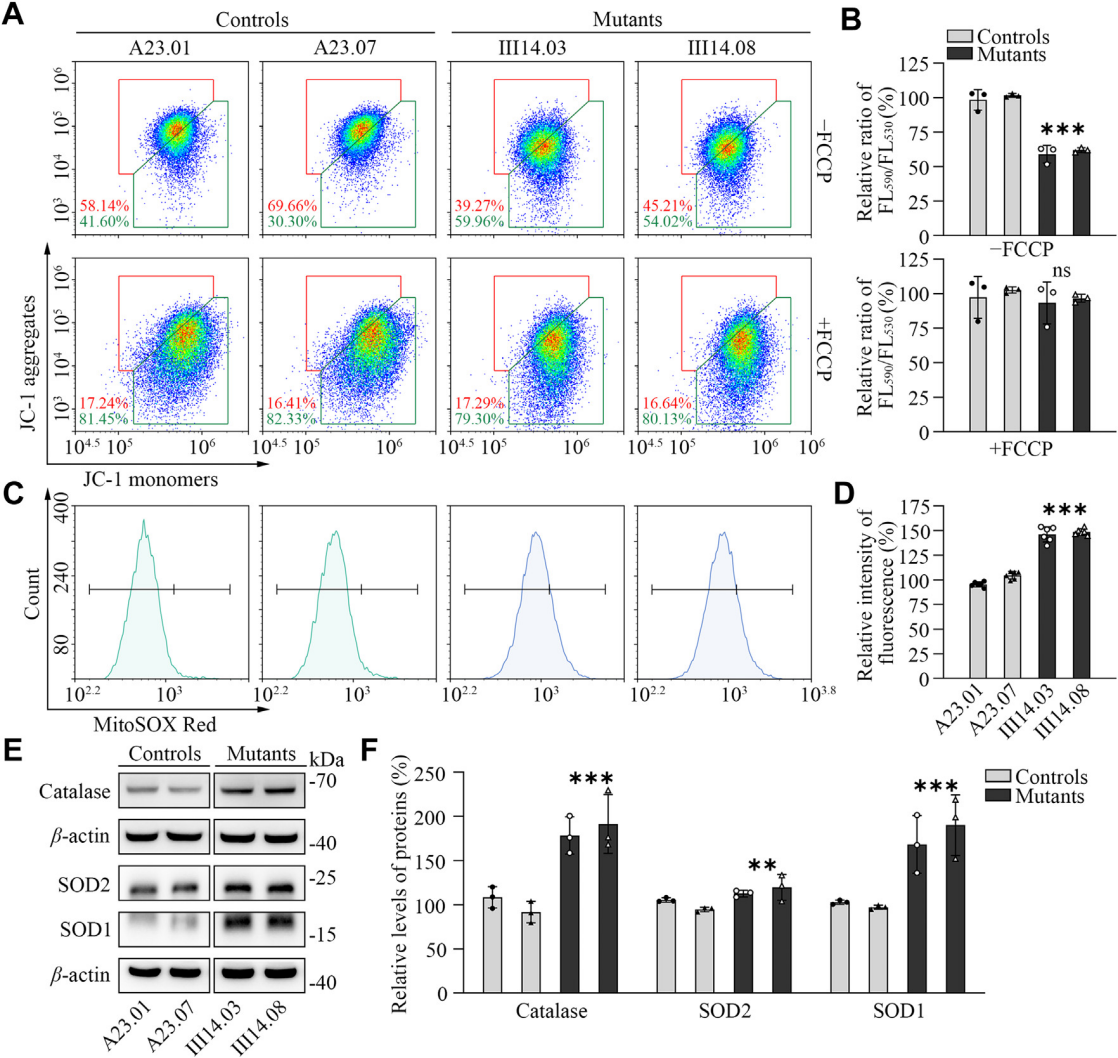
**Assays for mitochondrial membrane potential and ROS production.***A*, mitochondrial membrane potential analysis. Represented flow cytometry images of mutant and control cell lines in the presence and absence of 10 mM FCCP. *B*, the relative ratios of JC-1 fluorescence intensities at excitation/emission of 490/530 nm and 490/590 nm in the absence and presence of 10 mM of FCCP. *C*, the rates of ROS generation by mitochondria in living cells from two mutants and two control cybrids were analyzed by a Novocyte flow cytometer (ACEA Biosciences) using the mitochondrial superoxide indicator MitoSOX-Red (5 mM). Flow cytometry histogram showing MitoSOX-Red fluorescence of various cell lines. *D*, the relative ratios of intensity. The average value of three independent determinations for each cell line is shown. *E*, Western blot analysis of anti-oxidative enzymes SOD1, SOD2 and catalase in six cell lines with *β*-actin as a loading control. *F*, quantification of SOD1, SOD2, and catalase. The data were based on three independent experiments for each cell line. Graph details and symbols are explained in the legend to Figure 1.

The mitochondrial membrane potentials (ΔΨm) were measured through the fluorescence probe JC-1 assay system in two mutant and two control cybrids (50). The ratios of fluorescence intensities of Ex/Em = 490/590 and 490/530 nm (FL_590_/FL_530_) were recorded to delineate the ΔΨm of each sample. The relative ratios of FL_590_/FL_530_ geometric mean between mutant and control cell lines were calculated to represent the level of ΔΨm. As shown in Figure 3, *A* and *B*, the ΔΨm of two mutant cybrids carrying the m.1555A>G mutation ranged from 51.6% to 63.7%, with an average of 60.5% of the mean values measured in two control cybrids. In contrast, the levels of ΔΨm in the mutant cybrids in the presence of FCCP were comparable with those of control cybrids.

### Overproduction of mitochondrial ROS

The levels of mitochondrial ROS among these cybrids were determined using a MitoSOX assay *via* flow cytometry (51). Geometric mean intensity was recorded to measure and delineate the rate of ROS of each sample. As shown in Figure 3, *C* and *D*, the levels of ROS generation in the mutant cybrids ranged from 134.5% to 154.0%, with an average of 147.4% (*p* < 0.001) of the mean values measured in the control cybrids under unstimulated conditions.

To further assess if the m.1555A>G mutation-induced mitochondrial ROS production affected the antioxidant systems, we examined the levels of three antioxidant enzymes: SOD2 in mitochondria, and SOD1 and catalase in the cytosol in the various cell lines (52, 53). As shown in Figure 3, *E* and *F*, significant increasing levels of these proteins were observed in the mutant cybrids, as compared with those in control cybrids. In particular, the average levels of catalase, SOD2, and SOD1 in two mutant cybrids carrying the m.1555A>G mutations were 184.9%, 123.4%, and 179.2% relative to the mean values measured in two control cybrids, respectively.

### Imbalance of mitochondrial dynamics

**Figure 4:**
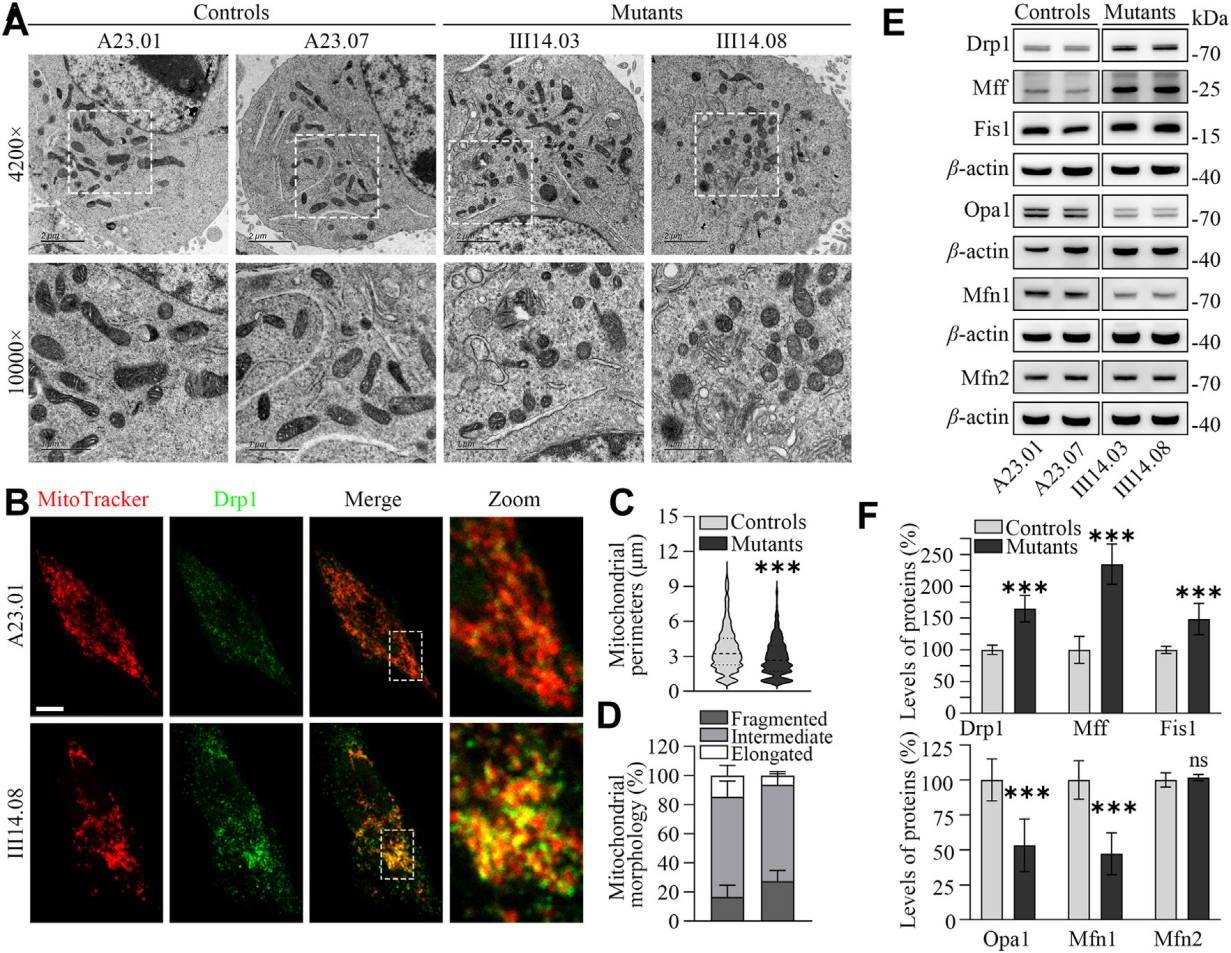
**Analysis of mitochondrial dynamics.***A*, mitochondrial morphology from mutant and control cybrids examined by transmission electron microscopy. *B*, immunofluorescence analysis. Mitochondrial morphologies in control (A23.01) and mutant (III14.08) cybrids were visualized by immunofluorescent staining with mitochondrial dye MitoTracker (*red*) and labeling with Drp1 antibody conjugated to Alex Fluor 488 (*green*) analyzed by confocal microscopy. Scale bars: 10 mm. *C*, quantification of mitochondrial perimeter (n ≥ 600 mitochondria counted per condition). *D*, quantification of mitochondrial morphologies, which were scored as follows: fragmented, mainly small and round; intermediate, mixture of round and shorter tubulated; and elongated, long and higher interconnectivity. The percentage of cells with indicated mitochondrial morphologies was determined as a percentage of the total number of cells counted (≥150 cells per experiment). n = 3 independent experiments. *E*, Western blot analysis for mitochondrial dynamics-related proteins: fusion-related proteins (Opa1, Mfn1, Mfn2) and fission-related proteins (Drp1, Fis1, Mff). *F*, quantification of mitochondrial dynamics proteins in the mutant and control cell lines. Graph details and symbols are explained in the legend in Figure 1.

Defective stability and activity of OXPHOS complexes caused by the m.1555A>G mutation may affect the mitochondrial dynamics, which is achieved through continual fusion and fission (54, 55). To evaluate the effect of m.1555A>G mutation on mitochondrial morphology and dynamics, we employed the transmission electron microscopy (TEM) and immunofluorescence using Drp1 along with MitoTracker staining. As illustrated in Figure 4, *A–D*, the mutant cells containing the m.1555A>G mutation exhibited abnormal mitochondrial morphology, characterized by significant increases in fragmented mitochondria and decreases in the elongated mitochondrial network, as compared to control cells. We then performed a Western blot analysis to measure the levels of fission-related proteins (Drp1, Mff and Fis1) and fusion-related proteins (Opa1, Mfn1, and Mfn2) in mutant and control cell lines. As shown in the Figure 4, *E* and *F*, mutant cybrids displayed elevating levels of fission-related proteins but reduced levels of fusion proteins, whereas the levels of Mfn2 in the mutant cybrids were comparable with those of control cybrids. As shown in the Figure 3*F*, the average levels of Drp1, Mff, Fis1, Opa1, Mfn1, and Mfn2 in two cybrids were 164.5%, 234.7%, 148.3%, 53.2%, 47.2%, and 101.7% of the average values measured in two cybrids respectively. These suggested that the m.1555A>G mutation led to an imbalance of mitochondrial dynamics toward fission.

### Impaired autophagy

**Figure 5:**
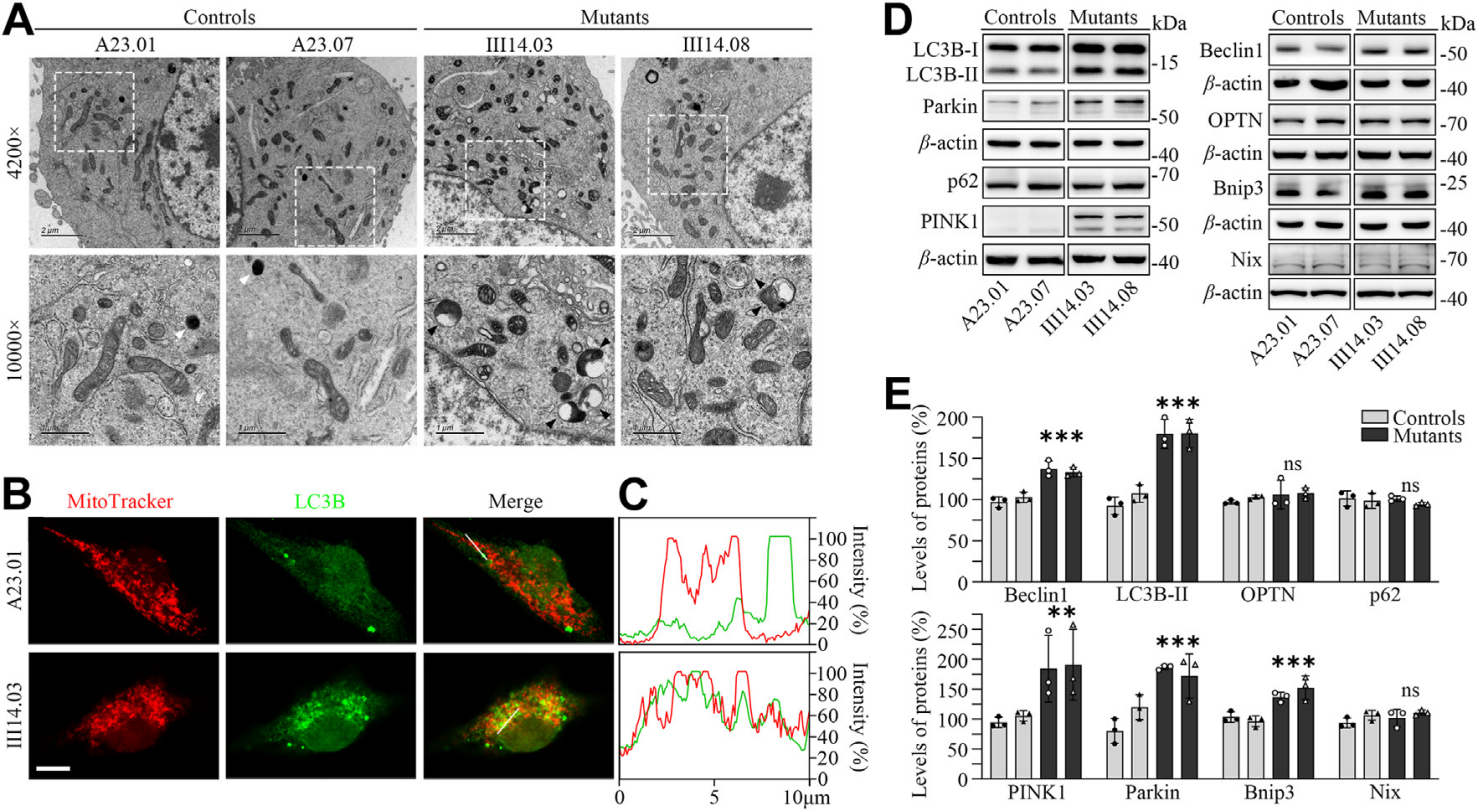
**Assessment of autophagy.***A*, autophagy from mutant and control cybrids assessed by transmission electron microscopy. Ultrathin sections were stained with uranyl acetate and alkaline lead citrate. Lysosomes (*white*) and autophagic vacuoles (*black*) were marked. 4200× and 10,000× magnifications were used, respectively. *B*, the distributions of LC3B from the control (A23.01) and mutant (III14.03) cell lines were visualized by immunofluorescent labeling with MitoTracker (*red*) and LC3B antibody conjugated to Alex Fluor 488 (*green*) analyzed by confocal microscopy. Scale bars: 10 mm. *C*, immunofluorescence colocalization analysis. Co-localization analysis of LC3B and mitochondria in the control (A23.01) and mutant (III14.03) cell lines. *D*, Western blot analysis for autophagic related proteins:LC3B, p62, Beclin1, OPTN, PINK1, Parkin, Bnip3, and Nix. *E*, quantification of autophagy-related proteins in the mutant and control cell lines. Graph details and symbols are explained in the legend to Figure 1.

Aberrant mitochondrial dynamics and membrane potentials due to mitochondrial dysfunctions may facilitate autophagy (56, 57, 58). The autophagy states of two mutant and two control cell lines were analyzed using transmission electron microscopy, immunofluorescence and Western blot assays. As shown in the Figure 5*A*, the mutant cybrids exhibited predominant accumulations of autophagic vacuoles, as compared with those in the control cybrids. Impaired autophagy was further evidenced by significantly increased levels of LC3B in the mitochondria, as compared with those in control cybrids (Fig. 5, *B* and *C*). We then measured the levels of autophagy-associated proteins: Beclin1, LC3B-II, OPTN and p62 (57, 58). As shown in the Figure 5, *D* and *E*, the average levels of Beclin1, LC3B-II, OPTN, and p62 in mutant cybrids were 135.4%, 180.1%, 107%, and 97.9% of the mean values measured in control cybrids, respectively. We then assessed the effect of m.1555A>G mutation on mitophagy, which is a specific form of autophagy that selectively removes damaged mitochondria by autophagosomes and their subsequent catabolism by lysosomes (58). Mitophagy can be classified based on the targeting signals present on damaged or excess mitochondria that trigger the process (58, 59, 60). This includes ubiquitin-dependent mitophagy, such as PARKIN-dependent pathways, as well as ubiquitin-independent or receptor-mediated mitophagy, which involves apoptosis-related proteins acting as mitophagy receptors or inhibitors (58, 59, 60). As shown in the Figure 5*D*, the mutant cybrids displayed markedly increased levels of PINK1 and Parkin involved in PARKIN-dependent pathway, mild elevated levels of Bnip3 but not Nip3-like protein X (Nix) involved in ubiquitin-independent pathways (58, 59, 60). As shown in the Figure 5*E*, the average levels of PINK1, Parkin, Bnip3, and Nix in mutant cybrids were 187.4%, 179.2%, 144.4%, and 106.2% of the mean values measured in control cybrids, respectively. These results suggested that the m.1555A>G mutation promoted both ubiquitin-dependent and ubiquitin-independent mitophagy.

### Upregulated intrinsic apoptosis

**Figure 6:**
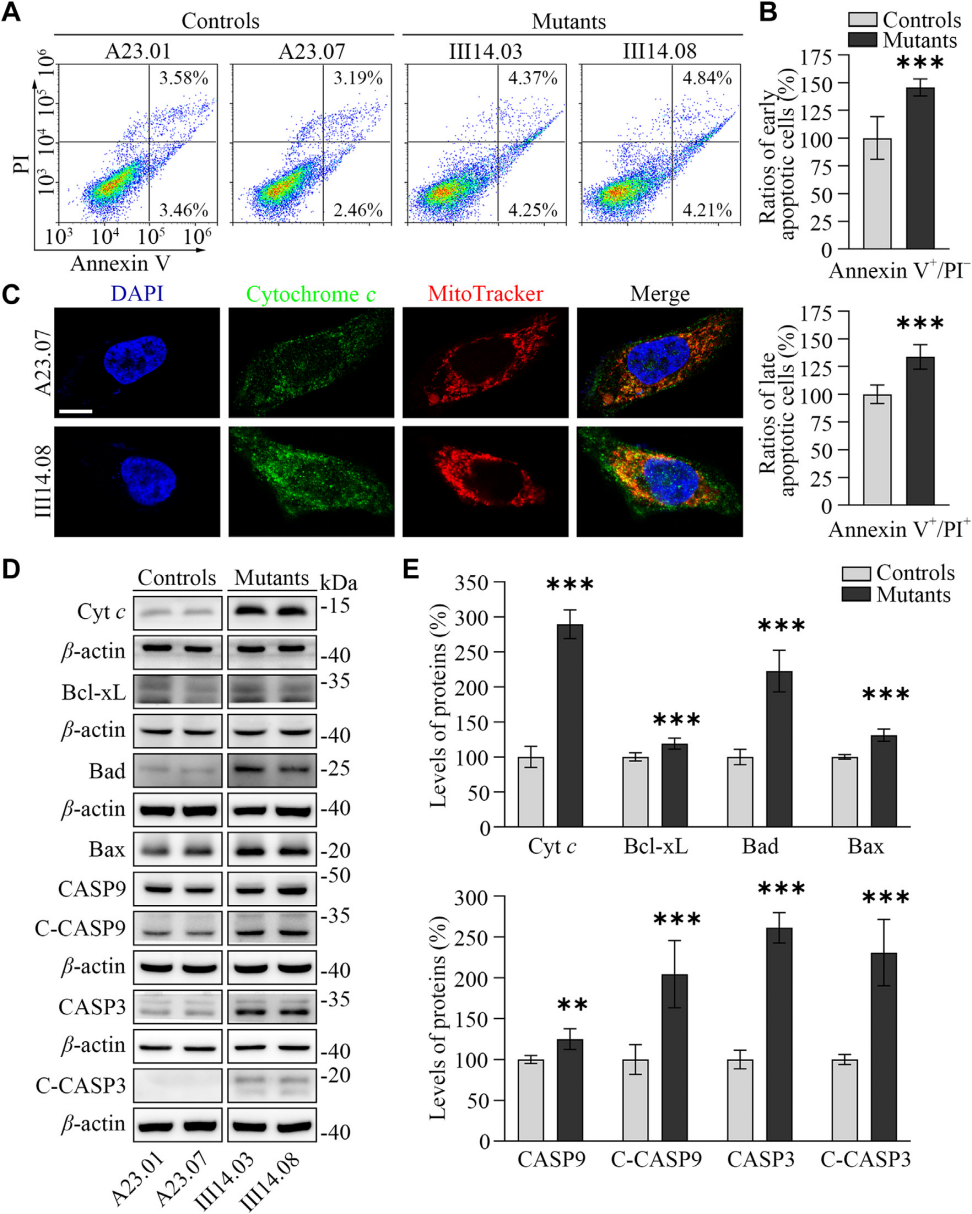
**Apoptosis assays.***A*, annexin V/PI apoptosis assay by flow cytometry. Cells were harvested and stained with Annexin V and 1 ml of propidium iodide. The percentage of Annexin V-positive cells were then assessed. *B*, relative Annexin V^+^/PI^–^ and Annexin V^+^/PI^+^ cells from various cell lines. Three independent determinations were done in each cell line. *C*, immunofluorescence analysis. The distributions of cytochrome c from control (A23.07) and mutant (III14.08) cybrids were visualized by immunofluorescent labeling with MitoTracker (*red*) and cytochrome *c* antibody conjugated to Alex Fluor 488 (*green*) analyzed by confocal microscopy. DAPI-stained nuclei were identified by their blue fluorescence. Scale bars: 10 mm. *D*, Western blot analysis of apoptosis-associated proteins: Cyt *c*, Bcl-xL, Bad, Bax, uncleaved/cleaved caspases nine and 3 with *β*-actin as a loading control. *E*, quantification of apoptosis-associated proteins. Three independent experiments were made for each cell line. Graph details and symbols are explained in the legend to Figure 1.

We assessed the impact of m.1555A>G mutation-induced deficiencies on intrinsic apoptosis using Annexin V/PI-based flow cytometry for apoptosis, immunofluorescence and Western blot assays. The annexin V^+^/PI^–^ and V^+^/PI^+^ indicate the early apoptotic cells, and late apoptosis or necrosis cells, respectively (4). As shown in the Figure 6, *A* and *B*, the average ratios of early apoptotic cells and late apoptotic cells in two mutant cybrids carrying the m.1555A>G mutation were 145.7% and 133.6% of the mean values measured in two control cybrids, respectively. As shown in Figure 6*C*, the immunofluorescence patterns of double-labeled cells with rabbit monoclonal antibody specific for cytochrome *c* and MitoTracker revealed significantly increased levels of cytochrome *c* in the mutant cybrids, compared with control cybrids. The higher levels of cytochrome *c* in the mutant cybrids than those in control cybrids were confirmed by Western blot analysis (Fig. 6, *D* and *E*). Furthermore, we examined the levels of one apoptosis inhibited protein (Bcl-xL) and six activated proteins (Bad, Bax, uncleaved/cleaved caspases nine and 3) in mutant and control cybrids (61, 62). As shown in the Figure 6, *D* and *E*, the average levels of Bcl-xL, Bad, Bax, uncleaved/cleaved caspases nine and three in two mutant cybrids were 118.9%, 222.5%, 131%, 124.9%, 204.4%, 261.1%, and 230.7% of mean values measured in two control cybrids, respectively. These results suggested that the m.1555A>G mutation facilitated the intrinsic apoptotic process.

## Discussion

In this study, we investigated the cellular consequences of deafness-associated m.1555A>G mutation at the mitochondrial level and interactions with the expression of certain nuclear genes required for organelle and cellular integrity. The m.1555A>G mutation induces the 1494-1555G-C base-pairing, yields a local conformational change in the A-site of 12S rRNA, and affects the efficiency and accuracy of codon-anticodon interaction, thereby perturbing mitochondrial translation (28, 29). An *in cellulo* [^35^S]-methionine pulse-labeling experiment revealed ∼35% average reductions in the rate of mitochondrial protein labeling in cybrid cell lines derived from matrilineal relatives from an Arabic-Israeli family carrying the m.1555A>G mutation (30). In the present study, the Western blot assay showed 33% average decreases of nine mtDNA-encoding polypeptides in the cybrids derived from a China family carrying the m.1555A>G mutation. In particular, the ND3, ND4L, ND5, CYTB, CO1, and CO2 in the mutant cybrids were significantly reduced, as compared with those in control cybrids. In fact, these mtDNA-encoded subunits function as precursors for the formation of new complexes, which necessitates the import and assembly of nucleus-encoded subunits with the help of assembly factors specific to complexes I, III, IV, and V (45, 46, 63). Thus, the impaired synthesis of these mtDNA encoding polypeptides arising from the m.1555A>G mutation likely contributed to the downregulation of nucleus-encoding subunits of complexes I (NDUFA10, and NDUFS1) and complex IV subunit (COXIV) but did not affect the expression of other nucleus-encoding subunits of complexes I, II, III and IV. Thus, both impaired synthesis of mtDNA-encoding ND3, ND4L, ND5, and CO2, and lower levels of nucleus-encoding NDUFA10, NDUFS1, and COXIV gave rise to aberrant assembly, instability activities of complexes I and IV rather than complexes II, III and V. The resultant respiratory deficiencies caused diminished mitochondrial ATP production and membrane potential, increased production of reactive oxygen species and the subsequent failure of cellular energetic processes.

To response to the m.1555A>G mutation-induced dysfunction, the mitochondria may reshape their integrity to regulate their quality control *via* fusion and fission processes as well as mitophagy (54, 55, 56). The effect of mitochondrial quality control by the m.1555A>G mutation was evidenced by the abnormality of mitochondrial morphologies, characterized by pronounced fragmentation and elongated network in the mutant cells. Especially, the cybrids bearing the m.1555A>G mutation revealed the upregulation of DRP1, FIS1 and MFN related to mitochondrial fission process but the downregulation of OPA, MFN1 but not in MFN2 involved in mitochondrial fusion process. These highlighted that the m.1555A>G mutation affected the mitochondrial quality control by an imbalance of mitochondrial dynamics through promoting fission. Alternatively, the m.1555A>G mutation promoted mitophagy by upregulating both the ubiquitin-dependent mitophagy, evidenced by increasing levels in the Pink and Parkin, and ubiquitination-independent mitophagy, supported by elevated expression of BNIP3 and NIX (59, 60, 64). These results were consistent with those in cells bearing a deafness-associated tRNA^Phe^ 593T>C or LHON-associated tRNA^Ala^ 5587T>C mutations (4, 22) and but contrast with the upregulation of ubiquitination-dependent mitophagy but downregulation of ubiquitination-independent mitophagy in the cells carrying the LHON-associated ND1 3460G>A or ND6 14484T>C mutation (65, 66). These discrepancies may reflect the defects in multiple polypeptides due to the 12S rRNA and tRNA mutations but only one defective polypeptide due to ND1 3460G>A or ND6 14484T>C mutation. This interplay between mitochondrial dynamics and mitophagy assures the homeostasis of cells.

The m.1555A>G mutation-induced deficiencies regulated the cell homeostasis *via* autophagy and apoptosis. In this study, the cells bearing the m.1555A>G mutation exhibited predominant accumulations of matured late autophagic vacuoles and marked increases in the levels of LC3, indicating the upregulation of autophagy (57). The m.1555A>G mutation-induced autophagy to break down and reuse old cell parts was consistent with increased autophagy in the cells carrying the tRNA^Met^ 4435A>G and tRNA^Phe^ 593T>C mutations (22, 43). Alternatively, the m.1555A>G mutation promoted the intrinsic apoptotic process for the removal of damaged cells. In this study, we demonstrated that the m.1555A>G mutation upregulated the apoptosis, evidenced by markedly increased levels of Annexin V intensity and release of cytochrome *c* into the cytosol. The release of cytochrome c promotes the activation of caspase-3, -7, and -9, which subsequently initiates cell death. The impact of m.1555A>G mutation on the intrinsic apoptotic process was further supported by increased levels of BAD and BCL2L. These findings demonstrated that the m.1555A>G mutation reshaped cell homeostasis by upregulating autophagy and apoptosis.

In summary, we demonstrated the impact of deafness-associated 12S rRNA 1555A>G mutation on the mitochondrial and cellular integrity contributing to the pathological process of hearing loss. The 12S rRNA 1555A>G mutation downregulated the expression of nuclear genes encoding complexes I and IV subunits and impaired the assembly and activities of OXPHOS complexes. The m.1555A>G mutation-induced deficiencies regulated the mitochondrial integrity by causing mitochondrial dynamic imbalance towards fission and elevating mitophagy. Finally, the m.1555A>G mutation-induced deficiencies reshape the cell homeostasis *via* promoting autophagy and intrinsic apoptosis. The broad effect of the m.1555A>G mutation on mitochondrial and cellular integrity may regulate various aspects of hearing function, thereby being critical for the pathogenesis of deafness. Thus, our findings may provide new insights into the pathophysiology of maternally inherited deafness arising from reshaping mitochondrial and cellular integrity due to 12S rRNA mutation.

## Experimental procedures

### Cell lines and culture conditions

Lymphoblastoid cell lines were generated from one hearing-impaired Chinese proband harboring the m.1555A>G mutation (WZD92 III-14), and one hearing normal control subject (A23) belonging to the same mtDNA haplogroup (B4) lacking the mutation (31). Immortalized lymphoblastoid cell lines were grown in RPMI 1640 medium (Corning) with 10% fetal bovine serum (FBS). The 143B.TK^–^ cell line was grown in DMEM (containing 4.5 mg of glucose and 0.11 mg pyruvate per ml), supplemented with 100 mg of BrdU per ml and 5% FBS. The mtDNA-less *ρ*^o^206 cell line, derived from 143B.TK^−^ was grown under the same conditions as the parental line, except for the addition of 50 mg of uridine/ml (44). Transformation by cytoplasts of mtDNA-less *ρ*^o^206 cells was performed by using immortalized lymphoblastoid cell lines, as detailed previously (30, 44). The cybrids derived from each donor cell line were analyzed for the presence and level of the m.1555A>G mutation and mtDNA copy numbers, as detailed previously (30). Two cybrids (III14.03 and III14.08) harboring the homoplasmic m.1555A>G mutation and two cybirds (A23.01, A23.07) lacking the mutation and similar mtDNA copy numbers were used for the biochemical characterization. All cybrid cell lines were maintained in the same medium as the 143B.TK^−^ cell line.

### Western blot analysis

Western blot analysis was performed as described previously (17, 67). Protein extracts (20–30 mg) obtained from cell lysates were electrophoresed using SDS-PAGE (10%–15% gels) and electroblotted onto a polyvinylidene difluoride (PVDF) membrane. The membrane was blocked by Tris-Buffered Saline and Tween20 (TBST) (150 mM NaCl, 10 mM Tris-HCl, pH 7.5, and 0.1% Tween 20) containing 5% (w/v) milk for 2 h and incubated with corresponding primary antibodies overnight at 4 °C. The antibodies used in this investigation are summarized in the Table S1. Horseradish peroxidase goat anti-mouse IgG and goat anti-rabbit IgG (Beyotime) were used as secondary antibodies, and protein signals were detected using the ECL system (CW-BIO). Quantification of density in each band was performed as detailed elsewhere (17).

### Blue native polyacrylamide gel electrophoresis assays

BN-PAGE was performed by isolating mitochondrial proteins from various cell lines, as detailed elsewhere (47, 48). Samples containing 30 mg of total mitochondrial proteins were separated on three to 12% Bis-Tris Native PAGE gel. The native gels were prewashed in cold water and then incubated with the substrates of complex I, complex II, complex IV, and complex V at room temperature, as detailed elsewhere (47, 48). After stopping the reaction with 10% acetic acid, gels were washed with water and scanned to visualize the activities of respiratory chain complexes.

### Measurements of oxygen consumption

The OCR in various cybrid cell lines was measured with a Seahorse Bioscience XF-96 extracellular flux analyzer (Seahorse Bioscience), as detailed previously (49). Cells were seeded at a density of 2 ×10^4^ cells per well on Seahorse XF96 polystyrene tissue culture plates (Seahorse Bioscience). Inhibitors were used at the following concentrations: oligomycin (1.5 mM), FCCP (0.8 mM), antimycin A (1.5 mM), and rotenone (3 mM), respectively.

### Assessment of mitochondrial membrane potential

Mitochondrial membrane potential was assessed with JC-1 Assay Kit-Microplate (Beyotime) according to the general manufacturer's recommendations with some modifications, as detailed previously (50, 67).

### Measurement of ROS production

ROS measurements were performed as detailed elsewhere (52, 67).

### Immunofluorescence analysis

Immunofluorescence experiments were undertaken as described elsewhere (43). Cells were cultured on cover glass slips (Thermofisher), fixed in 4% formaldehyde for 15 min, permeabilized with 0.2% Triton X-100, blocked with 5% FBS for 1 h, and immune-stained with DRP1, Cytochrome *c*, LC3B antibodies overnight at 4 °C, respectively. The cells were then incubated with Alex Fluor 594 goat anti-rabbit IgG, Alex Fluor 488 goat anti-rabbit IgG and Alex Fluor 488 goat anti-mouse IgG (Thermofisher), stained with 4′, 6-diamidino-2-phenylindole (DAPI; Thermofisher) for 15 min, and mounted with Fluoromount (Sigma-Aldrich). Cells were examined using a confocal fluorescence microscope (Olympus Fluoview FV1000) with three lasers (Ex/Em = 550/570, 492/520, and 358/461 nm).

### Transmission electron microscopy

The cell lines were washed with PBS and fixed in 2.5% glutaraldehyde in phosphate buffer (0.1 M, pH 7.0) for 4 h and post-fixed with 1% OsO_4_ in phosphate buffer (0.1 M, pH 7.0) for 2 h. The samples were dehydrated with increasing concentrations of ethanol (30, 50, 70, 80, 90, 95, and 100) and transferred to absolute acetone for 20 min. After placing in 1:1 mixture of absolute acetone and the final Spurr resin (SPI-CHEM, 02690-AB) mixture for 1 h at room temperature, the samples were transferred to 1:3 mixture of absolute acetone and the final resin mixture for 3 h and to the final Spurr resin mixture for overnight. Electron photomicrographs were taken from the ultrastructures of cells under transmission electron microscopy (Hitachi, H-7650).

### Annexin V/PI apoptosis assay by flow cytometry

For discrimination of apoptotic and non-apoptotic cells by Annexin V/PI staining. Cells were harvested and stained with Annexin V and 1 ml of propidium iodide (PI) (Themo Fisher Scientific) according to the manufacturer’s instruction. Each sample was detected by NovoCyte (ACEA Biosciences) and analyzed using NovoExpress software (4).

### Statistical analysis

All statistical analyses were performed using GraphPad Prism (version 9.00) for statistical analysis to compare outcomes using a two-tailed unpaired Student’s *t* test. *p* values of less than 0.05 were considered to be statistically significant. ∗*p* < 0.05; ∗∗*p* < 0.01; ∗∗∗*p* < 0.001; ns, not significant.

## Data availability

The authors declare that all relevant data of this study are available within the article or from the corresponding author (gminxin88@zju.edu.cn) upon reasonable request.

## Supporting information

This article contains supporting information.

## Conflict of interest

The authors declare that they have no conflicts of interest with the contents of this article.
