# Supplementary material for: Deafness-associated mitochondrial 12S rRNA mutation reshapes mitochondrial and cellular homeostasis
Source: J Biol Chem. 2024 Dec 22;301(2):108124. doi: 10.1016/j.jbc.2024.108124 (PMC11791119; doi:10.1016/j.jbc.2024.108124)
Supplement: Supporting Information [file mmc1.pdf]

Supplemental Table S1. List of antibodies used in the study.

REAGENT or RESOURCE	SOURCE	IDENTIFIER
Rabbit monoclonal anti-SDHC	Abcam	Cat#ab155999
Rabbit monoclonal anti-SDHAF1	Abcam	Cat#ab185222
Mouse monoclonal anti-P62	Abcam	Cat#ab56416
Rabbit polyclonal anti-BECN1	Abcepta	Cat#AP1818b
Mouse monoclonal anti-PINK1	Abcepta	Cat#AW5456
Rabbit polyclonal anti-ND3	Abclonal	Cat#A17969
Rabbit polyclonal anti-ND4L	Abclonal	Cat#A17971
Rabbit polyclonal anti-ND6	Abclonal	Cat#A17991
Rabbit polyclonal anti-NDUFA10	Abclonal	Cat#A10123
Rabbit polyclonal anti-NDUFS2	Abclonal	Cat#A12858
Rabbit polyclonal anti-CYB	Abclonal	Cat#A17966
Rabbit polyclonal anti-CO1	Abclonal	Cat#A17889
Rabbit polyclonal anti-NDUFAF1	Abclonal	Cat#A15835
Rabbit polyclonal anti-Catalase	Abclonal	Cat#A11780
Rabbit polyclonal anti-SOD1	Abclonal	Cat#A12537
Rabbit polyclonal anti-SOD2	Abclonal	Cat#A19576
Rabbit monoclonal anti-LC3B	Abclonal	Cat#A19665
Rabbit polyclonal anti-PRKN	Abclonal	Cat#A0968
Rabbit monoclonal anti-TOM20	Abclonal	Cat#A19403
Rabbit monoclonal anti-β-Actin	Abclonal	Cat#AC038
Rabbit monoclonal anti-DRP1	Cell Signaling	Cat#8570
Rabbit monoclonal anti-OPA1	Cell Signaling	Cat#80471
Rabbit polyclonal anti-Bcl-xL	Cell Signaling	Cat#2762
Rabbit polyclonal anti-Caspase-3	Cell Signaling	Cat#9662
Rabbit polyclonal anti-Caspase-9	Cell Signaling	Cat#9502
Rabbit polyclonal anti-ND5	Proteintech	Cat#55410-1-AP
Rabbit polyclonal anti-NDUFS1	Proteintech	Cat#12444-1-AP
Rabbit polyclonal anti-SDHB	Proteintech	Cat#10620-1-AP
Rabbit polyclonal anti-UQCRC2	Proteintech	Cat#14742-1-AP
Rabbit polyclonal anti-UQCRFS1	Proteintech	Cat#18443-1-AP
Rabbit polyclonal anti-CO2	Proteintech	Cat#55070-1-AP
Rabbit polyclonal anti-COXIV	Proteintech	Cat#11242-1-AP
Rabbit polyclonal anti-COX5A	Proteintech	Cat#11448-1-AP
Rabbit monoclonal anti-ATP6	Proteintech	Cat#68442-1-Ig
Rabbit polyclonal anti-ATP8	Proteintech	Cat#26723-1-AP
Rabbit polyclonal anti-ATPB	Proteintech	Cat#17247-1-AP
Rabbit polyclonal anti-ATP5C1	Proteintech	Cat#10910-1-AP
Rabbit polyclonal anti-BCS1L	Proteintech	Cat#60212-1-Ig
Rabbit polyclonal anti-COX16	Proteintech	Cat#19425-1-AP

**REAGENT or RESOURCE**	**SOURCE**	**IDENTIFIER**
Rabbit polyclonal anti-ATPAF1	Proteintech	Cat#18016-1-AP
Mouse monoclonal anti-MFF	Proteintech	Cat#66527-1-Ig
Rabbit polyclonal anti-FIS1	Proteintech	Cat#10956-1-AP
Rabbit polyclonal anti-MFN1	Proteintech	Cat#13798-1-AP
Rabbit polyclonal anti-MFN2	Proteintech	Cat#12186-1-AP
Rabbit polyclonal anti-OPTN	Proteintech	Cat#10837-1-AP
Mouse monoclonal anti-CYTC	Proteintech	Cat#66264-1-Ig
Mouse monoclonal anti-BAD	Proteintech	Cat#67830-1-Ig
Rabbit polyclonal anti-BAX	Proteintech	Cat#50599-2-Ig
Rabbit polyclonal anti-BNIP3	Sangon Biotech	Cat#D121876
Rabbit polyclonal anti-NIX	Santa Cruz	Cat#sc-166332
